# Catechol glucosides act as donor/acceptor substrates of glucansucrase enzymes of *Lactobacillus reuteri*

**DOI:** 10.1007/s00253-017-8190-z

**Published:** 2017-03-03

**Authors:** Evelien M. te Poele, Vincent Valk, Tim Devlamynck, Sander S. van Leeuwen, Lubbert Dijkhuizen

**Affiliations:** 1 Microbial Physiology Research Group, Groningen Biomolecular Sciences and Biotechnology Institute (GBB), University of Groningen, Groningen, The Netherlands; 2 Centre for Industrial Biotechnology and Biocatalysis, Department of Biochemical and Microbial Technology, Faculty of Bioscience Engineering, Ghent University, Coupure Links 653, 9000 Ghent, Belgium

**Keywords:** Glucansucrase, *Lactobacillus reuteri*, Catechol glucosides, Acceptor reaction, Glucosyl donor

## Abstract

Previously, we have shown that the glucansucrase GtfA-ΔN enzyme of *Lactobacillus reuteri* 121, incubated with sucrose, efficiently glucosylated catechol and we structurally characterized catechol glucosides with up to five glucosyl units attached (te Poele et al. in Bioconjug Chem 27:937–946, 2016). In the present study, we observed that upon prolonged incubation of GtfA-ΔN with 50 mM catechol and 1000 mM sucrose, all catechol had become completely glucosylated and then started to reappear. Following depletion of sucrose, this glucansucrase GtfA-ΔN used both α-d-Glc*p*-catechol and α-d-Glc*p*-(1→4)-α-d-Glc*p*-catechol as donor substrates and transferred a glucose unit to other catechol glycoside molecules or to sugar oligomers. In the absence of sucrose, GtfA-ΔN used α-d-Glc*p*-catechol both as donor and acceptor substrate to synthesize catechol glucosides with 2 to 10 glucose units attached and formed gluco-oligosaccharides up to a degree of polymerization of 4. Also two other glucansucrases tested, Gtf180-ΔN from *L. reuteri* 180 and GtfML1-ΔN from *L. reuteri* ML1, used α-d-Glc*p*-catechol and di-glucosyl-catechol as donor/acceptor substrate to synthesize both catechol glucosides and gluco-oligosaccharides. With sucrose as donor substrate, the three glucansucrase enzymes also efficiently glucosylated the phenolic compounds pyrogallol, resorcinol, and ethyl gallate; also these mono-glucosides were used as donor/acceptor substrates.

**Electronic supplementary material:**

The online version of this article (doi:10.1007/s00253-017-8190-z) contains supplementary material, which is available to authorized users.

## Introduction

Glucansucrases are large multi-domain extracellular enzymes that are classified into the glycoside hydrolase family 70 (GH70) (Lombard et al. 2014). They only have been detected in lactic acid bacteria, in members of the genera *Leuconostoc*, *Streptococcus*, *Lactobacillus*, and *Weissella*. Glucansucrases cleave the glycosidic bond of sucrose and transfer the glucose moiety to an acceptor substrate with release of the fructosyl moiety. Depending on the acceptor substrate, glucansucrases catalyze three types of reactions, i.e., polymerization, hydrolysis, and the acceptor reaction. In polymerization, the main reaction of glucansucrases, the glucose moiety is coupled to a growing chain of α-glucan oligo- and polysaccharides. Depending on the glucansucrase enzyme, the α-glucan products differ in linkage type, the type and degree of branching, and molecular mass. One kind, or a mixture of (α1→2), (α1→3), (α1→4), or (α1→6) glycosidic linkages, may be found. The glucansucrase GtfA-ΔN of *Lactobacillus reuteri* 121 (Kralj et al. 2002) produces a reuteran-type branched α-glucan containing 58% (α1→4) and 42% (α1→6) glycosidic linkages and up to 14% branching (van Leeuwen et al. 2008b). When water is the acceptor substrate, glucansucrases hydrolyze sucrose into glucose and fructose. In the acceptor reaction the glucose unit is transferred to other carbohydrates than a growing α-glucan chain, or to other hydroxyl-group containing organic molecules (Cote and Robyt 1982; Leemhuis et al. 2013). Glucansucrase enzymes are interesting biocatalysts for the glycosylation of small organic molecules, because they are promiscuous towards a wide range of acceptor substrates (Monsan et al. 2010; Leemhuis et al. 2012). Using inexpensive sucrose as glycosyl donor, glucansucrase enzymes glucosylate, for instance, phenolic compounds such as catechol (Meulenbeld and Hartmans 2000; Devlamynck et al. 2016; te Poele et al. 2016) and hydroquinone (Seo et al. 2009; Devlamynck et al. 2016), primary alcohols (Seibel et al. 2006; Devlamynck et al. 2016), and l-ascorbic acid (Kim et al. 2010). Glycosylation may change the physico-chemical and biological properties of molecules, enhance bioavailability of antibiotics, or improve stability of molecules against auto-oxidation (Desmet et al. 2012). Recently, we characterized a series of catechol glucosides produced by GtfA-ΔN, after incubation with catechol and sucrose (te Poele et al. 2016). Catechol glucosides up to a degree of polymerization (DP) of 5, with different combinations of (α1→4) and (α1→6) linkages, were structurally characterized using 1D/2D ^1^H NMR spectroscopy, and compared to α-glucan structures synthesized by GtfA-ΔN from sucrose only. Interesting similarities were found, but also clear structural differences. A branched catechol glucoside was identified and a catechol glucoside with two successive (α1→6) glycosidic linkages, structures that were not found when GtfA-ΔN was incubated with sucrose only (te Poele et al. 2016).

In this study, we analyzed the glucosylation of phenolic compounds by the GtfA-ΔN, and by two related *L. reuteri* glucansucrase enzymes Gtf180-ΔN and GtfML1-ΔN (both having 78% amino acid identity to GtfA-ΔN (Kralj et al. 2004a)), in more detail. All three enzymes can efficiently synthesize mono-glucosylated catechol (catG1), pyrogallol, resorcinol, and ethyl-gallate, and subsequently reuse these compounds as glucosyl donor. Incubation of GtfA-ΔN with mono-glucosylated catechol synthesized catechol glucosides with up to 10 glucose units. GtfA-ΔN could also use α-d-Glc*p*-(1→4)-α-d-Glc*p*-catechol (cat^4^G2) as donor substrate. Gtf180-ΔN could use α-d-Glc*p*-(1→3)-α-d-Glc*p*-catechol (cat^3^G2) and α-d-Glc*p*-(1→6)-α-d-Glc*p*-catechol (cat^6^G2) as donor substrates. GtfML1-ΔN was unable to use any of the catG2 substrates as glucosyl donor.

## Materials and methods

### Purification of recombinant glucansucrase enzymes

Recombinant, N-terminally truncated GtfA-ΔN of *L. reuteri* 121 (Kralj et al. 2004b), Gtf180-ΔN of *L. reuteri* 180 (Kralj et al. 2004a), and GtfML1-ΔN of *L. reuteri* ML1 (Kralj et al. 2004a) were produced and purified as described previously (Kralj et al. 2004b; Meng et al. 2014). The purity of the enzymes was checked by SDS-PAGE, and enzyme concentrations were measured by absorbance at 280 nm using a NanoDrop 2000 spectrophotometer (Isogen Life Science, De Meern, The Netherlands).

### Standard reaction buffer

All enzymatic reactions were performed at 37 °C in reaction buffer containing 25 mM sodium acetate (pH 4.7) and 1 mM CaCl_2_, unless stated otherwise.

### Enzyme activity assays with sucrose as both glucosyl donor and acceptor substrate

To confirm activity of the glucansucrase enzymes after purification, enzyme activity assays were done in reaction buffer containing 100 mM sucrose and 0.125 mg/mL purified protein. For this, samples of 100 μL were taken every 30 s for 4 min and directly inactivated by adding 20 μL of 1000 mM NaOH for 30 min. The inactivated samples were diluted two times in deionized water, and from 10 μL of the diluted samples, the glucose and fructose were quantified enzymatically by monitoring the reduction of NADP as described previously (Mayer 1987). The release of fructose corresponds to the total enzyme activity, the release of glucose corresponds to the hydrolytic activity, and the amount of fructose minus the amount of glucose corresponds to the transglycosylation activity (van Geel-Schutten et al. 1999). One unit (U) of enzyme is defined as the amount of enzyme required for producing 1 μmol fructose monosaccharide or catechol (glucoside) per min. Accordingly, the activities of GtfA-ΔN, Gtf180-ΔN, and GtfMLI-ΔN were 26.7, 12.3, and 9.5 U/mg purified enzyme, respectively.

To determine the activity of GtfA-ΔN on sucrose as both glucosyl donor and acceptor substrate, enzyme activity assays were done with six different sucrose concentrations ranging from 3.1 to 100 mM, in reaction buffer and 0.125 mg/mL GtfA-ΔN, and performed as described above. Kinetic parameters were calculated by non-linear regression of the Michaelis-Menten equation with SigmaPlot v12.0.

### Enzyme activity assays with catechol glucosides as both glucosyl donor and acceptor substrate

α-d-Glc*p*-catechol (catG1) and α-d-Glc*p*-(1→4)-α-d-Glc*p*-catechol (cat^4^G2) synthesized by GtfA-ΔN (te Poele et al. 2016), and α-d-Glc*p*-(1→3)-α-d-Glc*p*-catechol (cat^3^G2) and α-d-Glc*p*-(1→6)-α-d-Glc*p*-catechol (cat^6^G2) synthesized by Gtf180-ΔN (Devlamynck et al. 2016), were isolated and purified using preparative normal-phase HPLC (NP-HPLC) as described previously (te Poele et al. 2016). GtfA-ΔN enzyme activity on catG1 was measured at six different catG1 concentrations ranging from 3.1 to 100 mM using 0.125 mg/mL GtfA-ΔN. The activities of GtfA-ΔN, Gtf180-ΔN, and GtfMLI-ΔN on catechol di-glucosides were measured at 100 mM cat^3^G2, cat^4^G2, and cat^6^G2 in reaction buffer and 1.25 mg/mL enzyme. Samples were taken every minute over a period of 9 and 12 min for catG1 and catG2, respectively, immediately inactivated by diluting a 10 μL sample in 250 μL of 80% methanol, and centrifuged for 2 min at 15,000×*g*. Total activity of GtfA-ΔN on catG1 was determined by measuring the release of catechol from catG1, and the activities of the enzymes on catG2 were measured by the decrease of catG2. Catechol-, catG1-, cat^3^G2-, cat^4^G2-, and cat^6^G2-concentrations were determined with NP-HPLC, using their corresponding calibration curves ranging from 1.56 to 100 mM.

### Enzymatic sucrose detection

Catechol acceptor reactions were heat inactivated at 100 °C for 15 min. Inactivated samples were diluted 100 times in deionized water. From the diluted sample, 10 μL was incubated in a total volume of 50 μL of 100 mM NaOAc (pH 5.5) with and without 2 μL invertase (BDH Chemicals; catalog number 390203D), to convert sucrose into glucose and fructose. The glucose and fructose were quantified as described above. The amount of remaining sucrose in the samples can be measured by subtracting the amount of glucose in the non-treated samples from the amount of glucose in the invertase-treated samples.

### Enzymatic glucosylation of catechol

To follow catechol glucosylation by GtfA-ΔN in time, incubation reactions were carried out in reaction buffer with 50 mM catechol (1,2-dihydroxybenzene, >99% pure, Sigma-Aldrich), 1000 mM sucrose, and 0.5 mg/mL purified GtfA-ΔN enzyme for 4 h. Samples were taken after 0, 30, 60, 80, 105, 135, and 240 min and were directly heat inactivated at 100 °C for 15 min. For NP-HPLC analysis, 10 μL samples were diluted in 250 μL of 80% MetOH and centrifuged for 2 min at 15,000×*g*.

### Use of catechol glucosides as donor substrates

The use of catechol glucosides as donor substrate was followed in time, using incubation reactions with 50 mM catG1 in reaction buffer and 0.250 mg/mL purified GtfA-ΔN enzyme. After 1 h, 200 mM sucrose was added and the mixture was incubated for an additional 1 h. Samples were taken at 0, 10, 30, and 60 min (just before addition of 200 mM sucrose), 70, 90, and 120 min, and immediately heat inactivated at 100 °C for 15 min. For NP-HPLC analysis, 10 μL sample was diluted in 250 μL of 80% MetOH and centrifuged for 2 min at 15,000×*g*. Twenty-four-hour incubations of reaction buffer containing 100 mM catG1 without enzyme or with 1.25 mg/mL GtfA-ΔN, Gtf180-ΔN, and GtfMLI-ΔN were analyzed with MALDI-TOF-MS. For NMR spectroscopy analysis of glucosidic linkages in GtfA-ΔN products from incubations with catG1 (100 mM) with and without sucrose (1000 mM), two 60-min incubations were done with reaction buffer containing 0.5 mg/mL GtfA-ΔN. NMR analysis was also performed on 60-min incubations of reaction buffer containing 0.5 mg/mL GtfA-ΔN and 100 mM cat^4^G2, 0.5 mg/mL Gtf180-ΔN and 100 mM cat^3^G2, and 0.5 mg/mL Gtf180-ΔN and 100 mM cat^6^G2. The catechol glucosides were purified from the reaction mixture by solid-phase extraction using Strata-X 33u Polymeric Reversed Phase columns (Phenomenex) and analyzed by 1D ^1^H NMR spectroscopy.

### Normal-phase HPLC (NP-HPLC)

All NP-HPLC analyses were performed on an UltiMate 3000 chromatography system (Thermo Fisher Scientific, Amsterdam, The Netherlands), equipped with an UltiMate 3000 VWD 3100 UV detector and an Endurance autosampler (Spark Holland, The Netherlands). Catechol and catechol glucoside peaks were detected at 276 nm. In all cases, a mobile phase of acetonitrile (solvent A) and 50 mM ammonium formate buffer, pH 4.4, (solvent B) was used. To follow in time the glucosylation and deglucosylation of catechol (glucosides) by GtfA-ΔN, reaction components were separated by injecting 40 μL diluted sample on a Luna NH_2_ chromatography column (250 mm × 10 mm, 10 μm particle size, Phenomenex) at a flow rate of 4.6 mL/min. Runs were started with a 5-min isocratic step of 90% solvent A followed by a linear gradient from 90 to 60% solvent A over 17 min. All other NP-HPLC analyses were performed on a Luna NH_2_ chromatography column (250 mm × 4.6 mm, 10 μm particle size, Phenomenex) at a flow rate of 1 mL/min. To follow the activity of GtfA-ΔN on catG1 over time, 20 μL sample was injected and runs were started with a 5-min isocratic step of 90% solvent A followed by a linear gradient from 90 to 60% solvent A over 17 min. Samples (20 μL) of the deglucosylation of different mono-glucosylated phenolics using GtfA-ΔN, Gtf180-ΔN, and GtfMLI-ΔN were started with a 2-min isocratic step of 80% solvent A followed by a linear gradient from 80 to 67% solvent A over 5 min and a final washing step of 3 min with 20% solvent A. Samples (20 μL) of the 60-min incubation of GtfA-ΔN with cat^4^G2 were started with a 2-min isocratic step of 80% solvent A followed by a linear gradient from 80 to 55% solvent A over 12 min.

### High-pH anion-exchange chromatography (HPAEC)

Incubations of 30 min of 1.25 mg/mL GtfA-ΔN and 1000 mM sucrose with and without 50 mM catechol were heat inactivated for 15 min and analyzed with HPAEC with pulsed amperometric detection (PAD). Samples were diluted 100 times in deionized water, and 25 μL of the dilutions was run on a CarboPac PA-1 column (250 mm × 4 mm, Dionex BV, Amsterdam, The Netherlands), using an ICS3000 Ion chromatograph (Dionex BV), equipped with an ICS3000 electrochemical detection cell for PAD with a gold working electrode (pulse potentials and durations: E1 = +0.1 V, 410 ms; E2 = −2.0 V, 20 ms; E3 = +0.6 V, 10 ms; E4 = −0.1 V, 60 ms). A 45-min linear gradient of 30–300 mM NaOAc in 100 mM NaOH (1 mL/min) was used.

### MALDI-TOF-MS

Experiments were performed on an Axima Performance™ mass spectrometer (Shimadzu Kratos Inc., Manchester, UK) equipped with a nitrogen laser (337 nm, 3 ns pulse width). Positive-ion mode spectra were recorded using the reflector mode at a resolution of at least 5000 Full Width at Half Maximum (FWHM) and acquired with software-controlled pulse-delayed extraction optimized for *m*/*z* 1500. Mass spectra were recorded from 1 to 5000 *m*/*z*, with ion-gate blanking set to 200 *m*/*z*. Samples were prepared by mixing 1 μL enzymatic reaction mixture with 1 μL of 10 mg/mL 2,5-dihydroxybenzoic acid in 70% ACN as matrix solution.

### NMR spectroscopy

One- and two-dimensional ^1^H and ^13^C NMR spectra, including ^1^H-^1^H and ^13^C-^1^H correlation spectra, were recorded at a probe temperature of 25 °C on a Varian Inova 600 spectrometer (NMR Department, University of Groningen, The Netherlands). One-dimensional 600-MHz ^1^H NMR spectra were recorded with 4800 Hz spectral width at 16k complex data points, using a WET1D suppression of the HOD signal. Two-dimensional ^1^H-^1^H COSY spectra were recorded in 256 increments of 4000 complex data points with a spectral width of 4800 Hz. Two-dimensional ^1^H-^1^H TOCSY spectra were recorded with MLEV17 mixing sequences with 50- and 150-ms spin-lock times. Two-dimensional ^13^C-^1^H HSQC and HMBC spectra were recorded with a spectral width of 4800 Hz in *t*_2_ and 10,000 Hz in *t*_1_ direction, using 128 increments of 2000 complex points. One-dimensional ^13^C NMR spectra were recorded in a 10,000 Hz spectral width, collecting 8000 transients of 32k complex points. All spectra were processed using MestReNov. 5.3 (Mestrelabs Research SL, Santiago de Compostela, Spain).

## Results

### Synthesis of catechol glucosides using the GtfA-ΔN enzyme

**Fig. 1:**
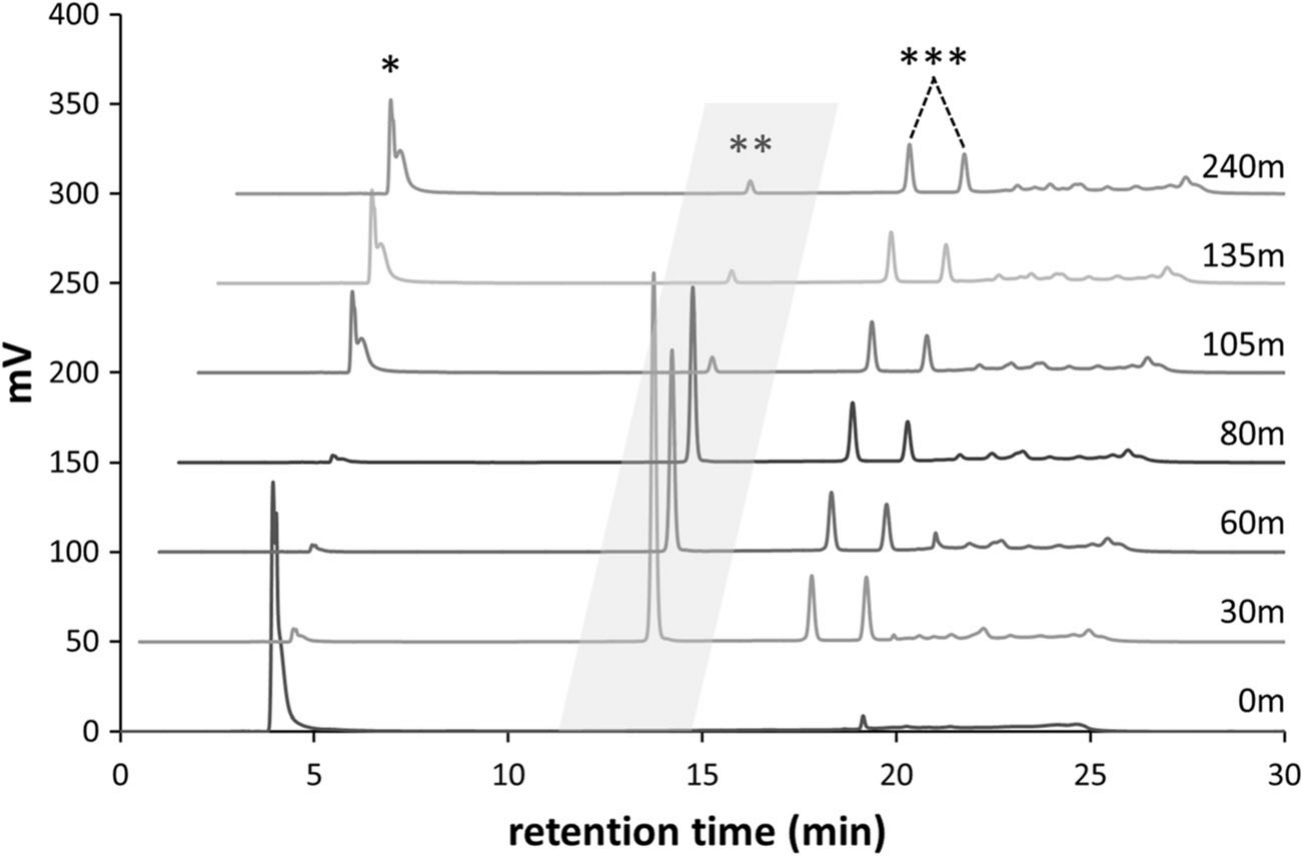
NP-HPLC product profiles (276 nm) of an incubation of 0.5 mg/mL GtfA-ΔN with 50 mM catechol and 1000 mM sucrose, incubated for 0, 30, 60, 80, 105, 135, and 240 min. *Asterisk* catechol peak at 4 min; *double asterisks* catG1 peak at 13.3 min in the *gray area*; *triple asterisks* cat^4^G2 and cat^6^G2 peaks at 17.3 and 18.7 min, respectively

**Fig. 2:**
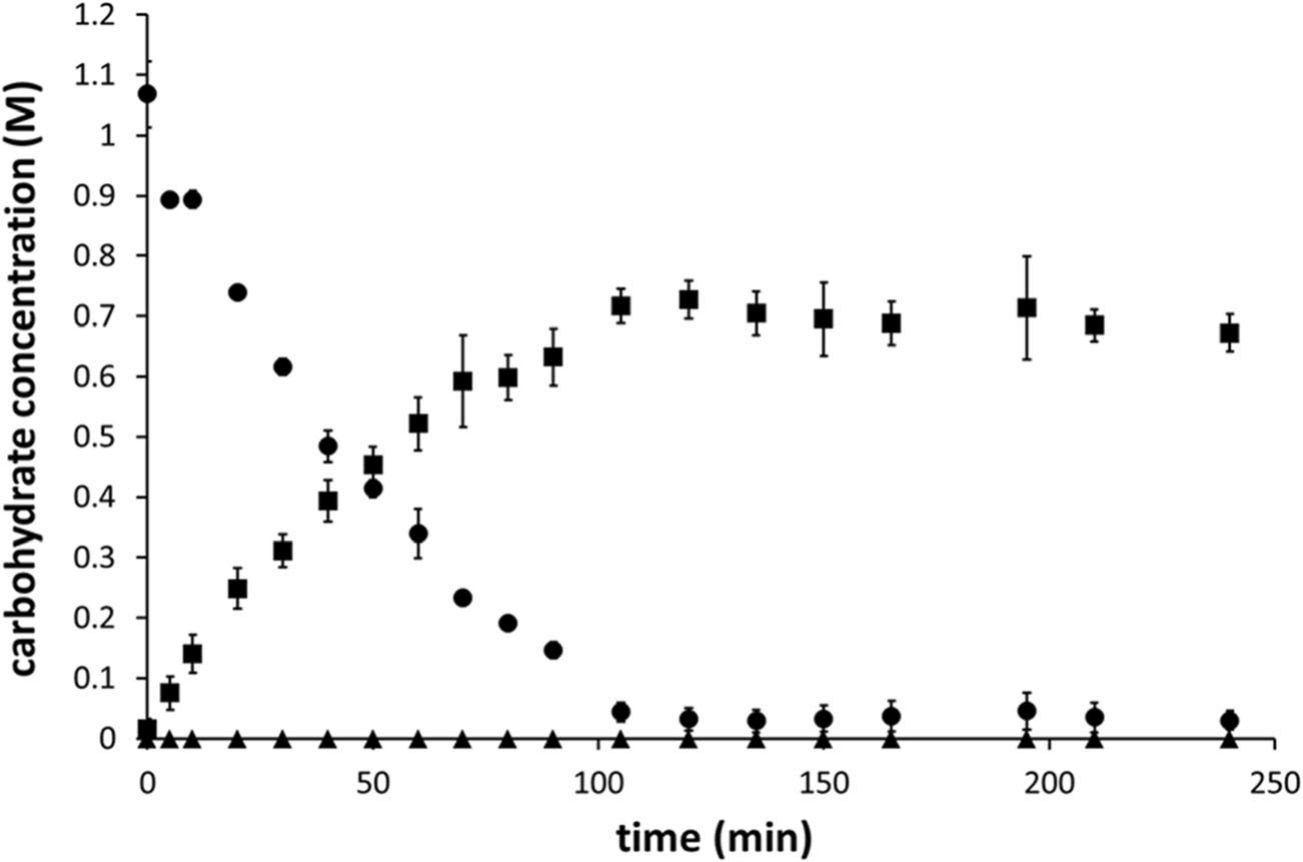
Graph showing the sucrose (*filled circle*) conversion and fructose (*filled square*) release during incubation of 0.5 mg/mL GtfA-ΔN with 50 mM catechol and 1000 mM sucrose for 240 min. No significant glucose (*filled triangle*) release was detected. The data represent the means of three independent enzymatic carbohydrate detection assays. *Error bars* are ±1 standard deviation

Previously, we showed that the transglycosylation/hydrolysis ratio of GtfA-ΔN and its overall transglycosylation activity increased with increasing sucrose concentrations (te Poele et al. 2016). A ~20-fold increase in transglucosylation activity was observed when the sucrose concentration was increased from 3.1 to 1000 mM, i.e., from 2.1 to 39.8 μmol/min/mg GtfA-ΔN. Therefore, 1000 mM sucrose was used as glucosyl donor in the catechol acceptor reactions. Incubations of 0.5 mg/mL GtfA-ΔN with 1000 mM sucrose and 50 mM catechol were followed in time (Figs. 1 and 2). After 30 min, all chatechol was converted, revealing multiple catechol glucoside product peaks detectable at 276 nm (Fig. 1). Interestingly, when sucrose was depleted at *t* = 105 min (Figs. 1 and 2), catechol reappeared, while the catG1 (te Poele et al. 2016) decreased. The catechol di-glucosides (te Poele et al. 2016) increased however, suggesting that transglycosylation occurred with catG1 as donor substrate. No detectable amounts of free glucose were found during these incubations (Fig. 2), suggesting that hydrolysis was negligible and all glucose from sucrose was used for synthesis of catechol glucosides and oligosaccharides (Supplemental material, Fig. S1). About 70% of the fructose was released (Fig. 2), showing that the other 30%, as fructose or as part of sucrose, served as acceptor molecule for glucosylation and was incorporated into leucrose and oligosaccharides (with terminal fructose units).

### GtfA-ΔN uses catG1 as glucosyl donor/acceptor substrate

**Fig. 3:**
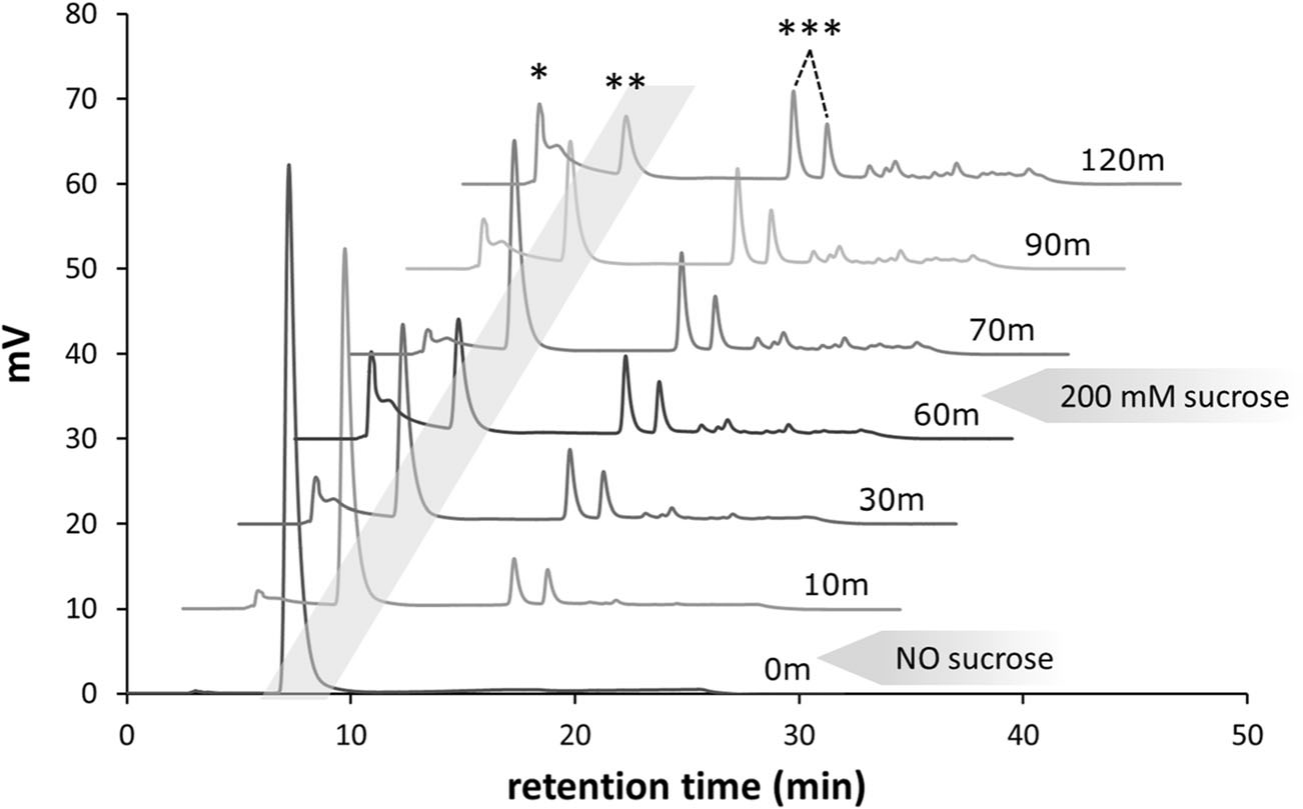
NP-HPLC product profiles (276 nm) of an incubation of 0.25 mg/mL GtfA-ΔN with 50 mM catG1 for *t* = 0, 30, and 60 min without sucrose and at *t* = 70, 90, and 120 min after the addition of 200 mM sucrose at 60 min. *Asterisk* catechol; *double asterisks* catG1 (in the *gray area*); *triple asterisks* catG2

To test whether GtfA-ΔN can use catG1 as glucosyl donor, 0.5 mg/mL GtfA-ΔN was incubated with 50 mM purified catG1 as sole substrate. As shown in Fig. 3, the amount of catG1 decreased during the 60-min incubation, catechol was released, and catechol glucosides with higher DP were produced. These data clearly show that catG1 acted both as donor and acceptor substrate; i.e., the glucose moieties of the catG1 donor substrate were transferred to catG1 acceptor molecules, resulting in formation of catechol glucosides of DP2 and higher. Addition of 200 mM sucrose to the 60-min catG1 incubation resulted again in glucosylation of catechol into catG1, as can be observed by the disappearance of catechol and the formation of catG1 after 10 min incubation (Fig. 3). After 70 min, sucrose was depleted (not shown) and the newly synthesized catG1 was again used as glucosyl donor resulting in the formation of catechol and larger catechol glucosides.

The transfer of glucose from catG1 to form multi-glucosylated catechol molecules was confirmed by MALDI-TOF-MS (Supplemental material, Fig. S2). After 24 h incubation of GtfA-ΔN with catG1, molecular masses up to a degree of polymerization of 10 glucose units (sodium adduct at 1755.18 *m*/*z*) could be detected. Besides catechol glucosides, oligosaccharides up to DP4 were synthesized (364.83, 526.84, and 688.98 *m*/*z*). The Gtf180-ΔN and GtfMLI-ΔN glucansucrase enzymes also were able to use catG1 as glucosyl donor, resulting in similar MALDI-TOF-MS product profiles with multi-glucosylated catechol (Supplemental material, Fig. S2). With sucrose as donor substrate, GtfA-ΔN, Gtf180-ΔN, and GtfMLI-ΔN also efficiently glucosylated the phenolic compounds resorcinol, pyrogallol, and ethyl gallate (data not shown) and used their mono-glucosides, resG1 (Devlamynck et al. 2016), pyrG1 (Supplemental material, Table S1 and Fig. S3), and etgaG1 (Supplemental material, Table S1 and Fig. S4) as glucosyl donor/acceptor substrates (Supplemental material, Fig. S5).

### GtfA-ΔN enzyme kinetics with sucrose and catG1 as glucosyl donor/acceptor substrates

**Fig. 4:**
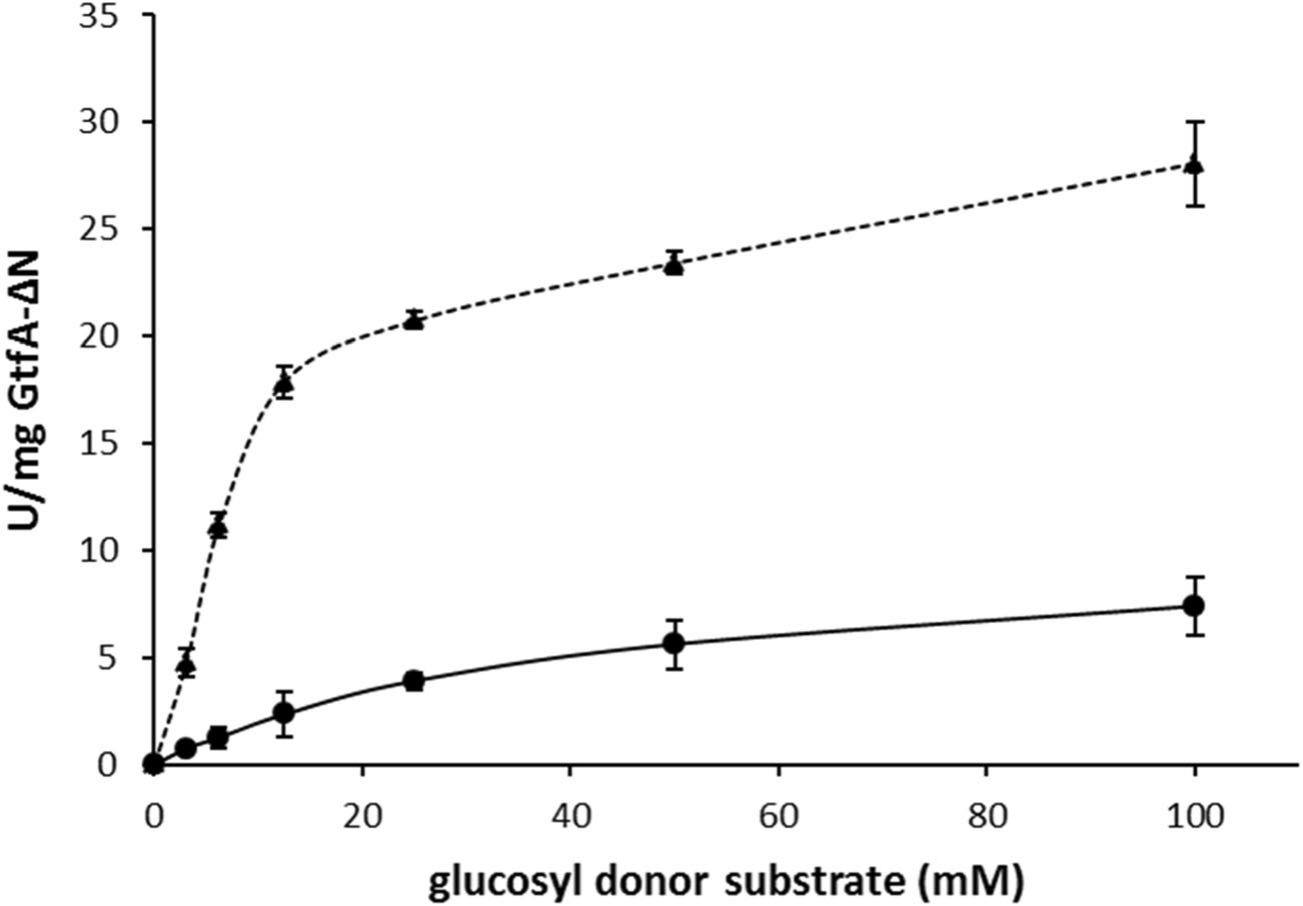
Effects of glucosyl donor substrate concentration, sucrose (*filled triangle*) and catG1 (*filled circle*), on initial GtfA-ΔN enzyme activity in reaction buffer and 0.125 mg/mL GtfA-ΔN at 37 °C and pH 4.7. *Error bars* are ±1 standard deviation

The kinetics of GtfA-ΔN with sucrose and catG1 as substrates was studied in more detail. For this, GtfA-ΔN was incubated with different concentrations sucrose and catG1. Compared to the initial reaction rate of GtfA-ΔN on 100 mM sucrose, the rate on 100 mM catG1 was ∼4-fold lower (Fig. 4). GtfA-ΔN displayed Michaelis Menten kinetics at sucrose and catG1 concentrations below 100 mM. The affinity (*k*_M_) values of GtfA-ΔN for sucrose and catG1 were determined as 9.7 and 44 mM, respectively. The maximum velocities (*V*_max_) were 28.5 and 10.5 U/mg GtfA-ΔN for sucrose and catG1, respectively.

**Fig. 5:**
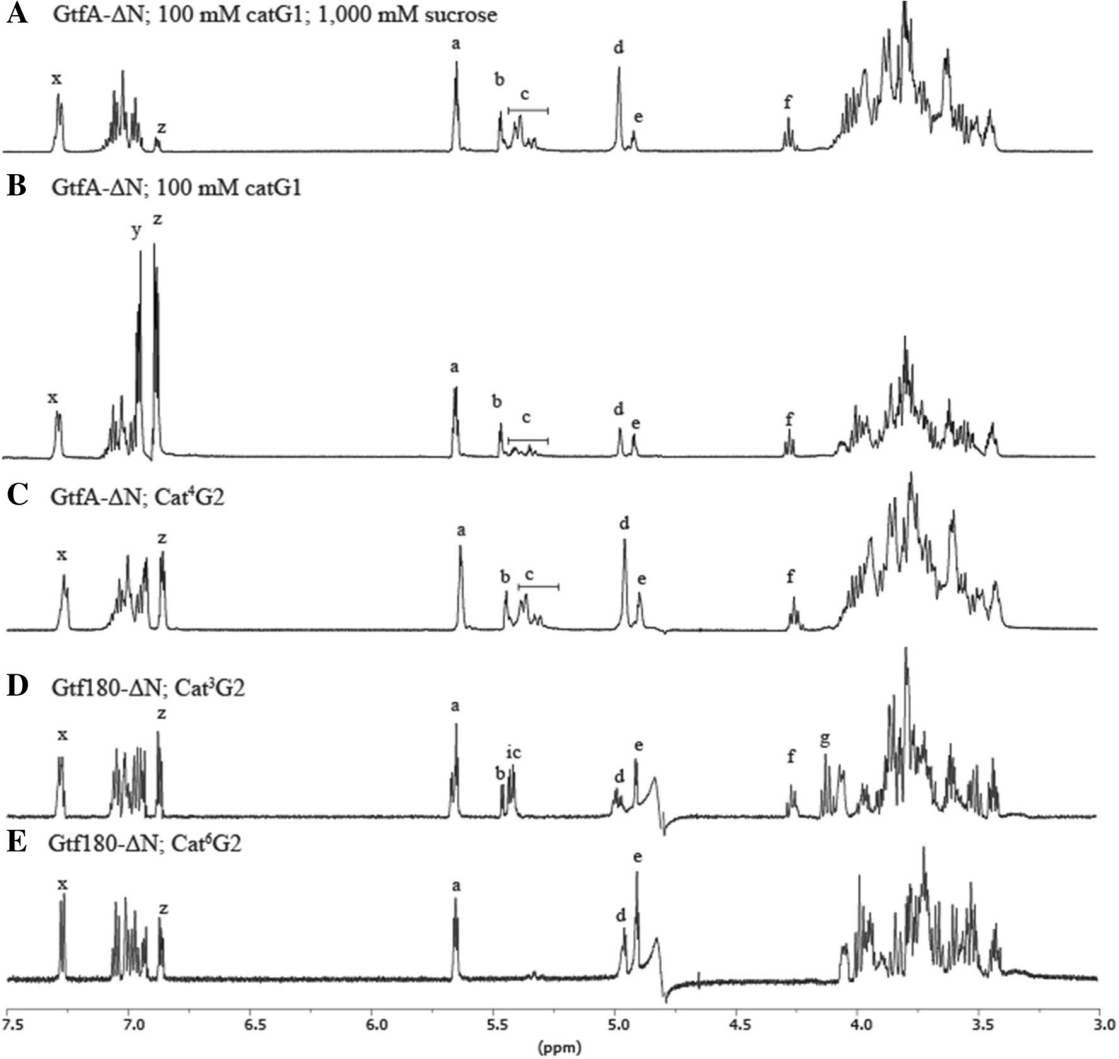
One-dimensional ^1^H NMR spectra of catechol glucosides produced by **a** GtfA-ΔN with 100 mM catG1 and 1000 mM sucrose, **b** GtfA-ΔN with 100 mM catG1, **c** GtfA-ΔN with cat^4^G2, **d** Gtf180-ΔN with cat^3^G2, and **e** Gtf180-ΔN with cat^6^G2. Peaks are marked with structural-reporter-group signals derived from te Poele et al. (2016) and Devlamynck et al. (2016), shown in Fig. 6

**Fig. 6:**
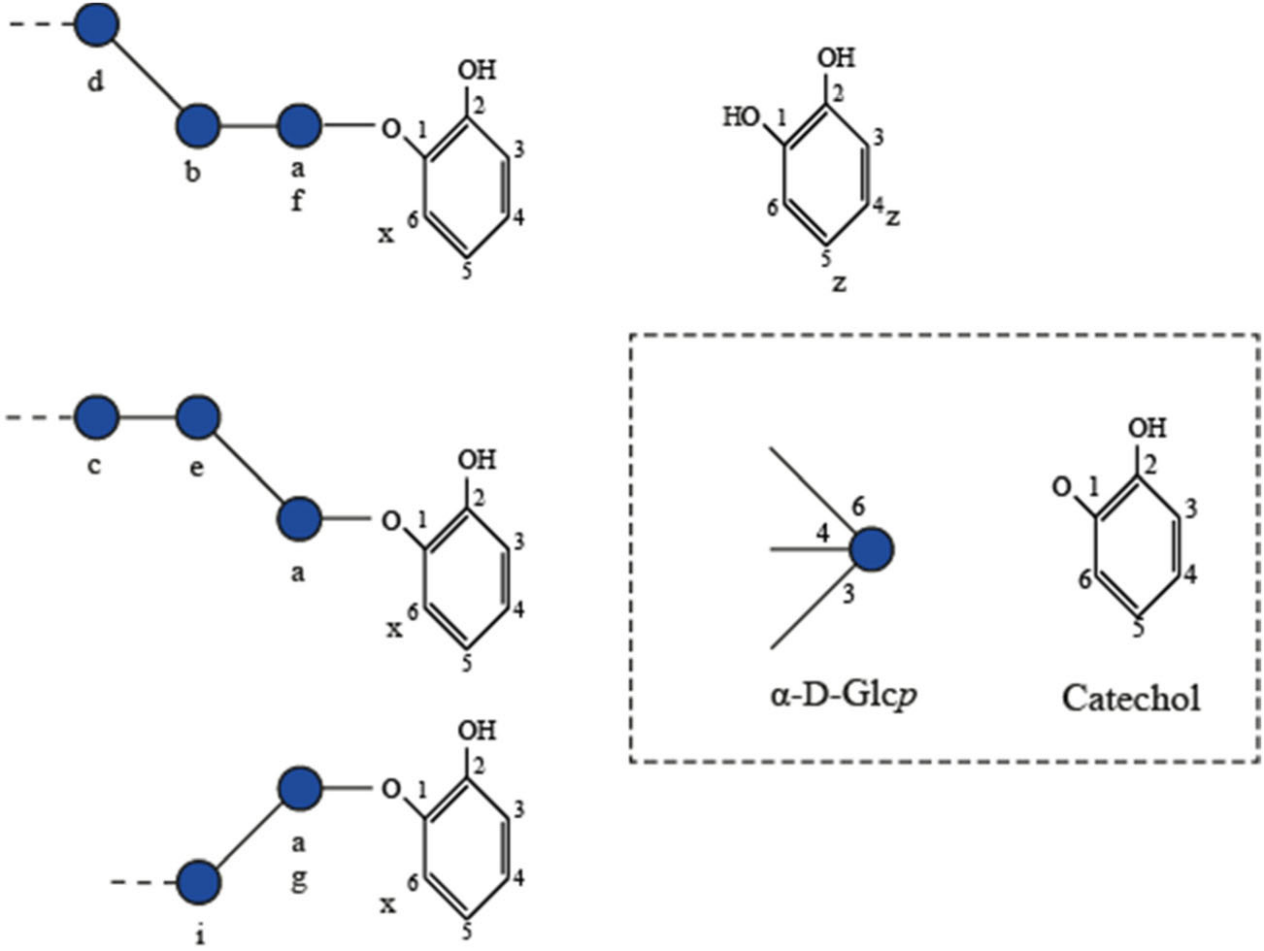
Overview of structural-reporter-group signals derived from te Poele et al. (2016) and Devlamynck et al. (2016) used for interpretation of 1D ^1^H NMR spectra

The GtfA-ΔN product spectrum with 100 mM catG1 as sole substrate was compared with the product spectrum using 100 mM catG1 in the presence of 1000 mM sucrose. The resulting catechol glucoside mixtures were analyzed by 1D ^1^H NMR spectroscopy (Fig. 5a, b) and NP-HPLC profiling (data not shown). Incubation with catG1 in the presence of sucrose resulted only in minor amount (7.3% based on NMR integrals *x* and 1/2 *z* of free catechol) (structural-reporter-group peaks z in Fig. 5a; Fig. 6), fitting with the clearly higher catalytic efficiency with sucrose as donor substrate (Fig. 4). With catG1 alone, a large amount (56.4% based on NMR integrals *x* and 1/2 *z*) of free catechol was formed (Fig. 5b). Both 1D ^1^H NMR spectra (Fig. 5a, b) showed structural-reporter-group signals a-f (Fig. 6), based on previous studies (Devlamynck et al. 2016; te Poele et al. 2016). When comparing the two 1D ^1^H NMR spectra, a notable difference in the ratio of peaks b and c was observed. Peaks in region b could be assigned to α-d-Glc*p*-(1→4) residues that are linked to the first glucose residue after catechol, irrespective of further substitution (Fig. 6). Peak f corresponds with a 4-substituted Glc residue, which is linked to the catechol residue. The intensity of peak f matches that of peak b in both 1D ^1^H NMR spectra, indicating that mainly cat^4^G2 core structures are formed. Peaks in region c correspond with α-d-Glc*p*-(1→4) elements in different configurations further down the chain at longer DPs. Peak e corresponds with α-d-Glc*p*-(1→6) residues linked to the first Glc residue after catechol, whereas peak d belongs to α-d-Glc*p*-(1→6) residues further down the chain in longer DPs. In the incubation with catG1 alone, peaks d and e have similar intensities, whereas the incubation with catG1 plus sucrose resulted in a much higher d peak. The (α1→4)/(α1→6) product ratios in the incubation with and without sucrose were 58:42 and 55:45, respectively.

### Use of di-glucosylated catechol as glucosyl donor/acceptor substrate

**Fig. 7:**
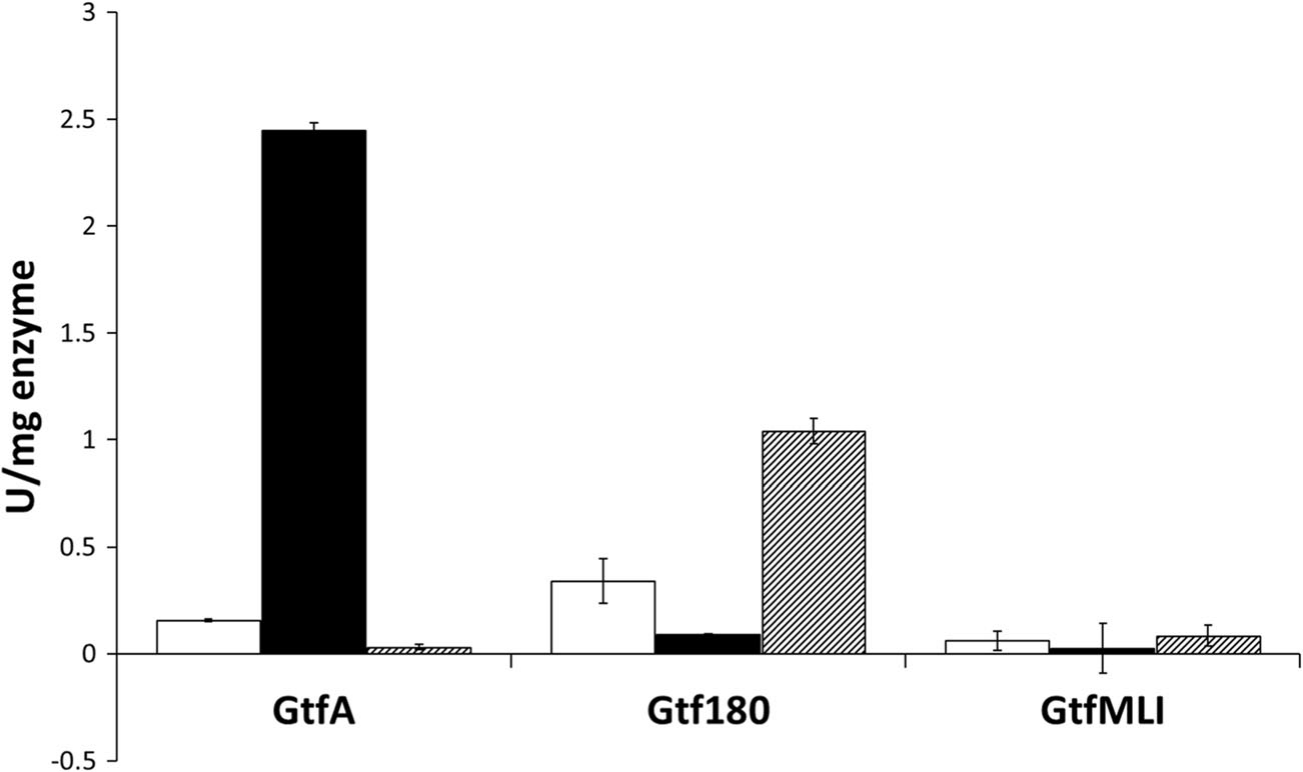
Effects of the glucosidic linkage type of di-glucosylated catechol on the initial enzyme activity in the reaction buffer and 1.25 mg/mL GtfA-ΔN, Gtf180-ΔN, and GtfMLI-ΔN at 37 °C and pH 4.7. The data represent the means of two independent enzyme activity assays; cat^3^G2 (*white bar*); cat^4^G2 (*black bar*); cat^6^G2 (*grey bar*). *Error bars* are ±1 standard deviation

Incubations with 100 mM cat^4^G2 as sole substrate showed that GtfA-ΔN also used di-glucosylated catechol as donor/acceptor substrate, although with a rate (2.3 U/mg GtfA-ΔN) that was ~75% lower than on catG1 (10.5 U/mg) and ~90% lower than on sucrose (28.5 U/mg) (Fig. 7). NP-HPLC analysis showed that cat^4^G2 was converted to catG1, free catechol, and catechol glucosides with three and more glucose units attached (Supplemental material, Fig. S6).

To test whether the glucosidic linkage type of catG1 had an effect on the transglucosylation rate of GtfA-ΔN, incubations were also performed with (α1→3)- and (α1→6)-linked catechol di-glucosides. GtfA-ΔN showed some activity on cat^3^G2 (0.16 ± 0.01 U/mg GtfA-ΔN), but showed no significant activity with cat^6^G2 (Fig. 7). Gtf180-ΔN used both cat^3^G2 and cat^6^G2 as donor/acceptor substrates, at 0.34 ± 0.04 and 1.04 ± 0.12 U/mg Gtf180-ΔN, respectively, but in this case, activity with cat^4^G2 was not significant (Fig. 7). GtfMLI-ΔN showed no significant activity with the three catechol di-glucosides (Fig. 7). 1D ^1^H NMR analysis of the Cat^4^G2-derived products following incubation with GtfA-ΔN (Fig. 5c) showed a product spectrum containing (α1→4) (peaks b, c, and f) and (α1→6) (peaks d and e) glucosidic linkages. Incubation of Cat^3^G2 with Gtf180-ΔN yielded products with (α1→3) (peaks i and g), (α1→4) (peaks b, c, and f), and (α1→6) (peaks d and e) glucosidic linkages (Fig. 5d). Incubation of Cat^6^G2 with Gtf180-ΔN resulted in a product spectrum with only (α1→6) (peaks d and e) glucosidic linkages (Fig. 5e).

## Discussion

In this study, we show that the glucansucrase GtfA-ΔN of *L. reuteri* 121 rapidly glucosylated catechol with sucrose as donor substrate. Upon sucrose depletion, the enzyme used several catechol glucosides as glucosyl donor/acceptor substrate. Especially mono-glucosylated catechol is an effective donor/acceptor substrate for GtfA-ΔN. This enzyme also efficiently used the mono-glucosides of several other phenolic compounds, such as pyrogallol and ethyl gallate as donor/acceptor substrates. Also the related *L. reuteri* enzymes, Gtf180-ΔN and GtfMLI-ΔN, use these phenolic mono-glucosides efficiently as donor/acceptor substrates. Notably, cat^4^G2 also functioned as donor substrate for GtfA-ΔN, while cat^3^G2 and cat^6^G2 were donor substrates for Gtf180-ΔN. The (α1→4)-linked di-glucosylated catechol was converted into catG1, free catechol, and catechol glucosides with three and more glucose units attached (Fig. 3). GtfA-ΔN first cleaves the glycosidic bond between the two glucose moieties of the cat^4^G2 donor substrate and then the bond between the first glucose and the catechol moiety of the resulting catG1. GtfA-ΔN appears unable to cleave of the two glucose units at once, since (besides catechol glucosides with an even number of glucose units) also catG1 and catechol glucosides with an uneven number of glucose units were detected (Supplemental material, Fig. S2). The donor binding pocket of the active site of GtfA-ΔN is most likely too small to bind sugars with more than one glucose unit, like maltose. The high-resolution crystal structure of the closely related Gtf180-ΔN (78% amino acid identity) with sucrose bound in the active site, shows that the donor binding cavity is blocked beyond subsite −1 where the glucose moiety of sucrose binds, and is therefore unable to bind longer oligosaccharides (Vujičić-Žagar et al. 2010). Recently, also the crystal structure of GtfA-ΔN was determined at 3.6 Å resolution by molecular replacement (Pijning et al. 2012). Superposition of the three-dimensional structure of GtfA-ΔN with the Gtf180-ΔN sucrose complex showed that the active site of GtfA-ΔN is almost identical to that of Gtf180-ΔN (Pijning et al. 2012). The three amino acids that are responsible for blocking the binding cavity in Gtf180-ΔN are conserved in GtfA-ΔN, and in all other family GH70 enzymes. This suggests that binding of a single glucose residue in subsite −1 is a general property of all glucansucrase enzymes in family GH70 (Vujičić-Žagar et al. 2010).

Compared to cat^4^G2, GtfA-ΔN showed minor activity on cat^3^G2, and had no significant activity on cat^6^G2. Gtf180-ΔN, on the contrary, uses both (α1→3)- and (α1→6)-linked di-glucosylated catechol as glucosyl donor, but not the (α1→4)-linked variant. The hydrolysis energies of the (α1→3), (α1→4), and (α1→6) linkages between the two glucoses of α-d-Glc*p*-(1→x) α-d-Glc*p*-catechol (catG2) are clearly lower than for sucrose, as evidenced by lower enzyme activities on the catG2 substrates, but apparently high enough to drive the glucansucrase-catalyzed donor reactions with these compounds. GtfA-ΔN thus uses cat^4^G2 as glucosyl donor, but as previously shown, maltose is not a donor substrate (Dobruchowska et al. 2013). The glucosylated phenolic compounds can be regarded as activated sugar donors, like *p*-nitrophenyl-α-glucoside (pNPG), since the aromatic ring functions as an excellent leaving group during the glucosyl transfer reaction (Andre et al. 2010). The presence of the catechol group most likely increases the hydrolysis energy of the (α1→4) glycosidic linkage to allow the transglycosylation reaction to occur. Previously, the free energies of enzymatic hydrolysis (ΔG^0^) of sucrose, maltose, and isomaltose have been experimentally determined as −26.5, −15.5, and −7.06 kJ/mol, respectively, using NP-HPLC and microcalorimetry (Goldberg et al. 1989; Tewari and Goldberg 1989). In future research, it would be interesting to calculate the free energies of hydrolysis of the newly formed catechol glucoside compounds. Apparently, the hydrolysis energy of the glucosidic linkages in catG2 is not the only factor that determines which catG2 the specific glucansucrase enzymes can use, since GtfA-ΔN can use cat^4^G2 as glycosyl donor, but Gtf180-ΔN cannot. Similarly, Gtf180-ΔN uses the (α1→6)-linked variant, but GtfA-ΔN does not. With sucrose only, GtfA-ΔN synthesizes a glucan with mostly (α1→4) and (α1→6) glucosidic bonds in a 58:42 ratio (van Leeuwen et al. 2008b), whereas Gtf180-ΔN prefers the synthesis of (α1→6) and (α1→3) glycosidic linkages in a 69:31 ratio (van Leeuwen et al. 2008a). The catG2 specificity of the glucansucrase enzymes thus appears to depend on both the hydrolysis energy of the glycosidic linkage of the substrate and the glucoside linkage specificity of the enzyme. Molecular dynamic studies are currently performed to get more insight in how mono- and di-glucosylated catechol are positioned in the donor binding cavity of the active site of GtfA-ΔN and Gtf180-ΔN.

It was assumed that glucansucrases have a limited promiscuity toward their glucosyl donor substrates (Andre et al. 2010). Only some early studies have reported the use of glucosyl donors other than sucrose by glucansucrases. Hehre and Suzuki (1966) found that dextransucrases of different *Leuconostoc mesenteroides* strains readily transferred the glucose moiety from sucrose to lactulose to produce lactulosucrose, but also that these enzymes utilized the glucose of lactulosucrose almost as efficiently as sucrose for the synthesis of dextran polymers. Glucansucrases from *Leuconostoc* and *Streptococcus* could also polymerize the glucosyl group of α-d-Glc*p*-fluoride (Genghof and Hehre 1972; Figures and Edwards 1976; Jung and Mayer 1981), α-d-Glc*p*-α-l-sorbofuranoside (*Streptococcus mutans*) (Mazza et al. 1975) and *p*-nitrophenyl α-d-glucopyranoside (pNPG) (Binder and Robyt 1983). With pNPG, the initial rate of release of *p*-nitrophenol from pNPG was ~100-fold lower than the initial rate of glucose incorporation into dextran from sucrose. Binder et al. (1983) showed that these glucansucrases also use several gluco-oligosaccharides, i.e., isomaltotriose, panose, maltotriose, and dextran as d-glucosyl donors and as acceptor substrates. When these gluco-oligosaccharides also acted as glucosyl acceptor substrates, disproportionation reactions occurred. From isomaltotriose, for example, isomaltose and isomaltotetraose were formed initially, and a series of isomalto-oligosaccharides were synthesized eventually.

Our findings show that glucansucrase enzymes from *L. reuteri* are more promiscuous towards glucosyl donor substrates as well. This may therefore be a general feature of glucansucrases and will have a severe impact on the final glucosylation yield of acceptor substrates, as observed with GtfA-ΔN and catechol upon prolonged incubation. Under the selected incubation conditions, the yield of catechol glucosylation was 100% after 30 min, but after 4 h the yield had decreased to ~50% due to de-glucosylation of catG1 and cat^4^G2 to catechol. The decrease in yield is even more pronounced when lower initial sucrose concentrations are used. In that case, with the same amount of enzyme and catechol, sucrose is depleted much earlier. At the time point of sucrose depletion, relatively more mono-glucosylated catechol that has not been further glucosylated to higher-DP glucosides is present, compared to incubations with higher sucrose concentrations. Since GtfA-ΔN can also use catG1 and cat^4^G2 as alternative glucosyl donors, more catechol will be reformed at lower sucrose concentrations, resulting in lower catechol conversion yields overall. Prolonged incubation beyond the time point of sucrose depletion thus may result in de-glucosylation of glycosides; reported yields for acceptor substrate reactions of glucansucrases may therefore be an underestimation.

We show that the studied glucansucrase enzymes efficiently glucosylate several phenolic compounds and that they use the produced glucosylated phenolics as glucosyl donor when sucrose is depleted. In case of catechol glucosides, the G2 products also served as glucosyl donors, but they were less efficient than the mono-glucosylated catechol.

## Electronic supplementary material

ESM 1 (PDF 2444 kb)
