# Supplementary material for: Catechol glucosides act as donor/acceptor substrates of glucansucrase enzymes of Lactobacillus reuteri
Source: Appl Microbiol Biotechnol. 2017 Mar 3;101(11):4495–505. doi: 10.1007/s00253-017-8190-z (PMC5442262; doi:10.1007/s00253-017-8190-z)
Supplement: Supplementary file 1 — (PDF 2444 kb) [file 253_2017_8190_MOESM1_ESM.pdf]

Supplementary material

Applied Microbiology and Biotechnology

Catechol glucosides act as donor/acceptor substrates of glucansucrase enzymes of *Lactobacillus reuteri*

Evelien M. te Poele[1], Vincent Valk[1], Tim Devlamynck[1,2], Sander S. van Leeuwen[1], and Lubbert Dijkhuizen[1]

[1] Microbial Physiology, Groningen Biomolecular Sciences and Biotechnology Institute (GBB), University of Groningen, Groningen, The Netherlands

[2] Centre for Industrial Biotechnology and Biocatalysis, Department of Biochemical and Microbial Technology, Faculty of Bioscience Engineering, Ghent University, Coupure Links 653, 9000 Ghent, Belgium

Corresponding Author:

Lubbert Dijkhuizen, Email: l.dijkhuizen@rug.nl, T: +31 503632150, F: +31 503632154

Structural analysis of pyrG1 and etgaG1.

1D ^{1}H NMR of **pyrG1** (Fig. S3) showed an anomeric signal at δ 5.426 (**A** H-1; $J_{1,2}$ 3.4 Hz), fitting an α-anomeric signal. The absence of a β-anomeric signal, suggests a non-reducing sugar, fitting a covalent coupling to pyrogallol. From the 2D ^{1}H-^{1}H correlation spectra (Fig. S3) all ^{1}H chemical shifts were determined (Table S1). The ^{1}H chemical shifts of the pyrogallol moiety were assigned based on 1D ^{1}H NMR intensity, showing 2 proton equivalent signal at δ 6.563 and one proton equivalent signal at δ 6.962 ppm. The occurrence of a single ^{1}H chemical shift for both **X** H-3 and H-5, suggests a symmetrical molecule, fitting a glucosylation on the pyrogallol O-1. Complete absence of ROESY correlations (not shown) between the **A** H-1 and **X** H-3 or H-5, supports this observation, in catechol and resorcinol glycosides (Devlamynck et al. 2016; te Poele et al. 2016) such correlations with protons on the carbon neighbouring the substituted O-1 were observed. Correlations in the 2D ^{13}C-^{1}H HSQC spectrum (Fig. S3) allowed assignment of ^{13}C chemical shifts of the glucose moiety, as well as the **X3**, **X4**, and **X5** carbons of the pyrogallol part. Carbons **X1**, **X2** and **X6** were determined by 1D ^{13}C NMR spectroscopy, combined by 2D ^{13}C-^{1}H HMBC spectroscopy (Fig. S3). The covalent coupling of glucose to pyrogallol O-1 is confirmed by the 3-bond HMBC correlation between **A** H-1 and **X** C-1.

In 1D ^{1}H NMR spectrum of **etgaG1** (Fig. S4) the anomeric signal at δ 5.507 (**A** H-1: $J_{1,2}$ 3.5 Hz) indicated a glucose moiety with an α-anomeric configuration. The absence of a β-anomeric signal indicates a non-reducing sugar, fitting a covalent coupling to ethylgallate. Using 2D ^{1}H and ^{13}C correlation spectra, combined with 1D ^{13}C NMR spectroscopy (Fig. S4), all ^{1}H and ^{13}C chemical shifts of **etgaG1** could be assigned (Table S1). Similar to the **pyrG1** structure, the single ^{1}H chemical shift observed for both **X** H-3 and H-5 (δ 7.078) indicating a symmetrical structure, suggests a substitution on **X** O-1. There was no ROESY coupling (not shown) observed between **A** H-1 and **X** H-3 or H-5, supporting the glucosylation on the **X** O-1. The coupling to ethylgallate O-1 is further confirmed by 3-bond HMBC correlation between **A** H-1 and **X** C-1.

References

Devlamynck T, te Poele EM, Meng X, van Leeuwen SS, Dijkhuizen L (2016) Glucansucrase Gtf180-ΔN of *Lactobacillus reuteri* 180: enzyme and reaction engineering for improved glycosylation of non-carbohydrate molecules. Appl Microbiol Biotechnol 100:7529–7539. doi: 10.1007/s00253-016-7476-x

te Poele EM, Grijpstra P, van Leeuwen SS, Dijkhuizen L (2016) Glucosylation of catechol with GTFA glucansucrase enzyme from *Lactobacillus reuteri*, using sucrose as donor substrate. Bioconjug Chem 27:937–946. doi: 10.1021/acs.bioconjchem.6b00018

Table

Table S1. ^{1}H and ^{13}C chemical shifts of **pyrG1** and **etgaG1** determined by 1D and 2D ^{1}H and ^{13}C NMR spectroscopy, measured at 25 °C. Precise ^{13}C chemical shifts were derived from high-resolution 1D ^{13}C NMR spectra. All values are relative to internal acetone ($\delta^{1}H$ 2.225; $\delta^{13}C$ 31.08)

	**pyrG1**		**etgaG1**	
	$\delta\ ^{1}H$	$\delta\ ^{13}C$	$\delta\ ^{1}H$	$\delta\ ^{13}C$
**A 1**	5.426	102.59	5.507	102.23
**A 2**	3.728	72.30	3.733	72.27
**A 3**	3.961	73.64	3.977	73.61
**A 4**	3.552	69.81	3.552	69.80
**A 5**	4.292	74.07	4.259	74.22
**A 6a**	3.800	60.99	3.795	61.00
**A 6b**	3.845	60.99	3.837	61.00
**X 1**	-	133.80	-	137.89
**X 2**	-	150.45	-	150.37
**X 3**	6.563	109.80	7.078	110.60
**X 4**	6.962	126.43	-	127.30
**X 5**	6.563	109.80	7.078	110.60
**X 6**	-	150.45	-	150.37
**X 7**	-	-	-	168.85
**X` 1**	-	-	4.315	63.17
**X` 2**	-	-	1.353	14.24

Figures

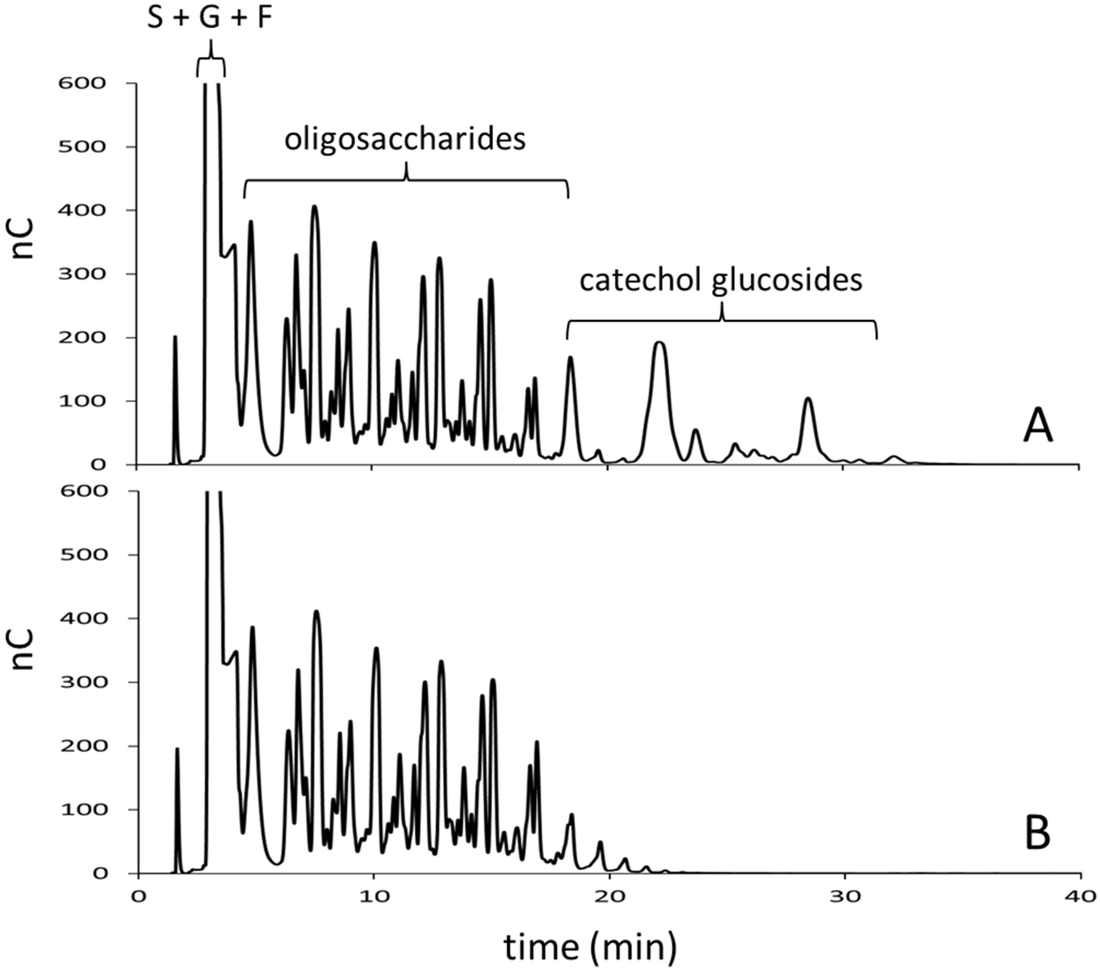

Fig. S1 HPAEC profiles of a 30 min incubation of 1.25 mg/mL GtfA-ΔN and 1,000 mM sucrose (A) with 50 mM catechol and (B) without catechol. S = sucrose; G = glucose; and F = fructose

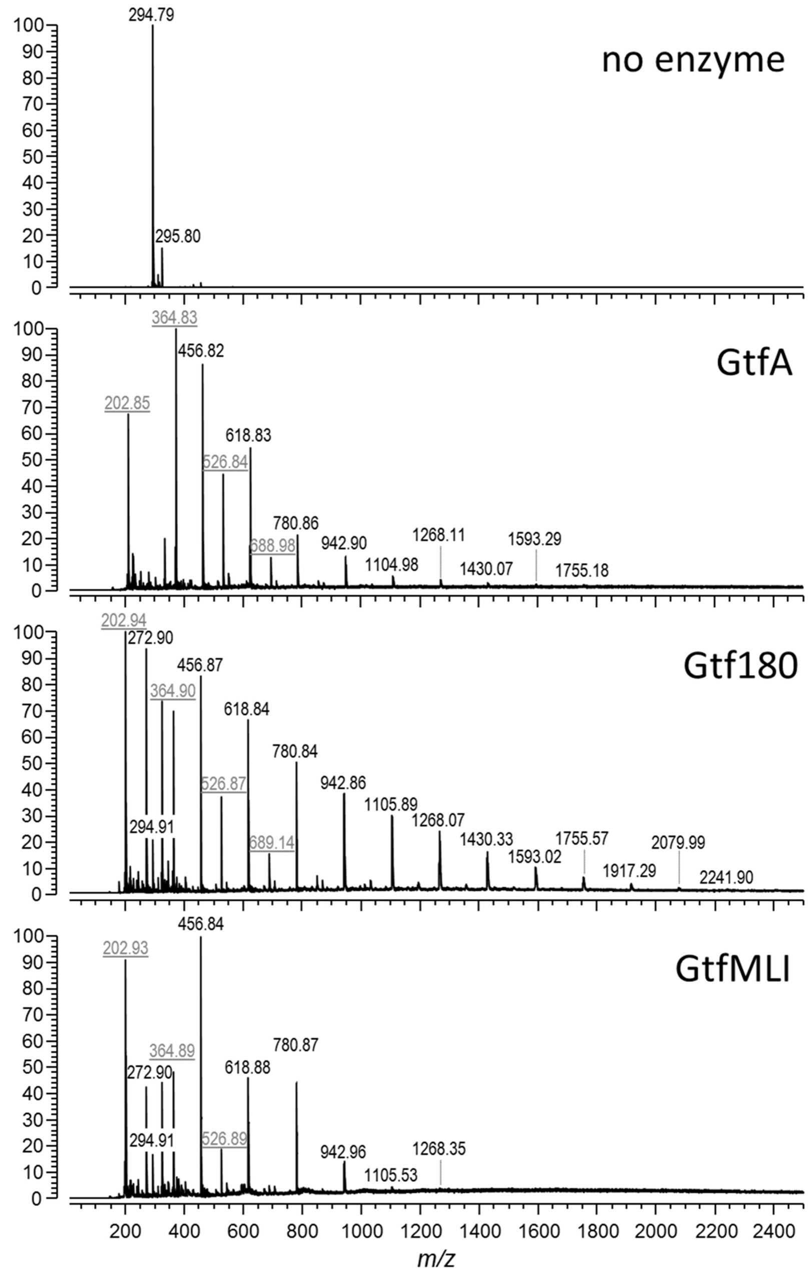

Fig. S2 MALDI-TOF MS mass spectra of 24 h incubations of 100 mM catG1 without enzyme and 1.25 mg/mL GtfA-ΔN, Gtf180-ΔN and GtfMLI-ΔN. The values of catechol glucosides are depicted in black and those of the gluco-oligosaccharides are depicted in grey and are underlined

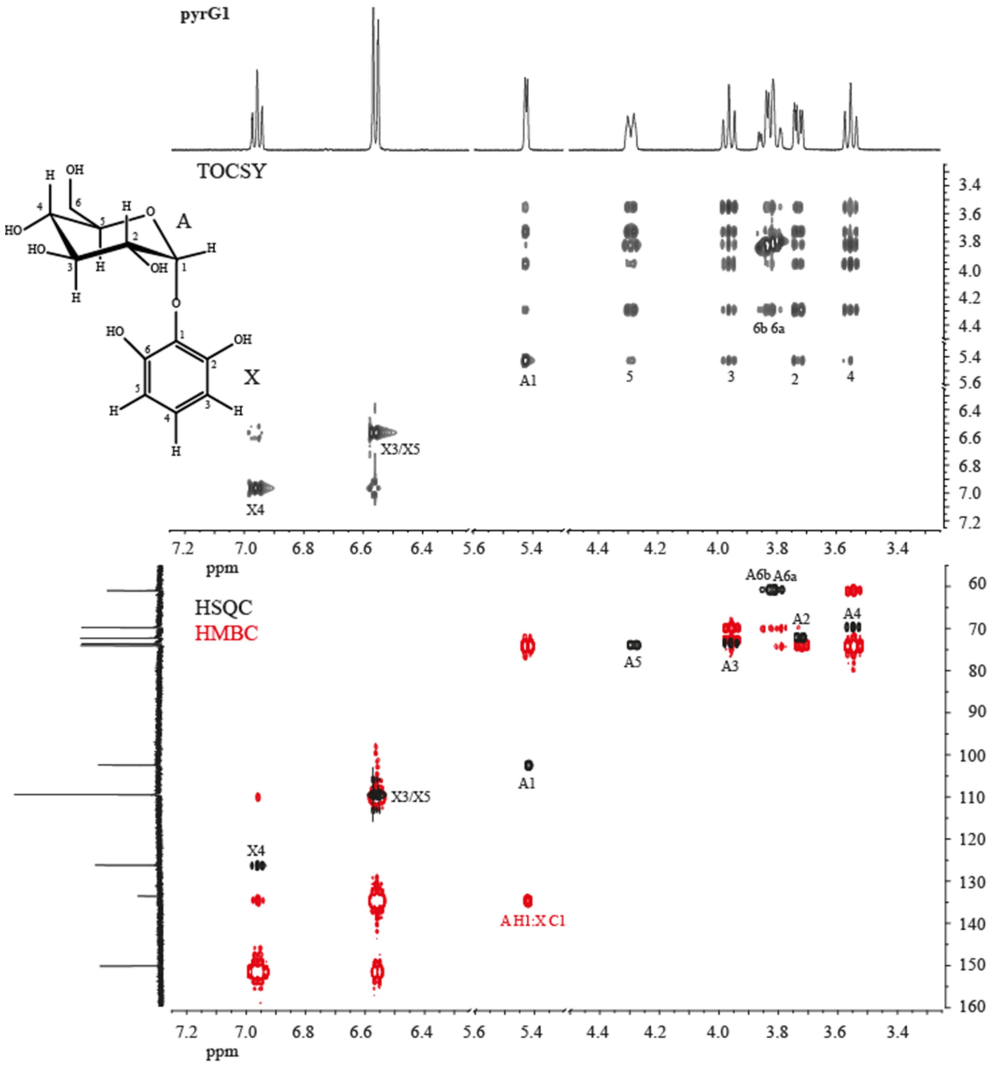

Fig. S3 1D ^{1}H NMR spectrum, 2D ^{1}H-^{1}H TOCSY (150 ms mixing time), 2D ^{13}C-^{1}H HSQC (black), and HMBC (red) spectra and 1D ^{13}C NMR spectrum of **pyrG1** structure. Peaks are marked in the spectrum, A1 signifies H-1 and/or C-1 of glucose residue A. Pyrogallol atoms are marked by X1-6. The relevant 3-bond HMBC correlation between A H-1 and X C-1 is marked. Peak numbers correspond with the atom numbers indicated in the structure

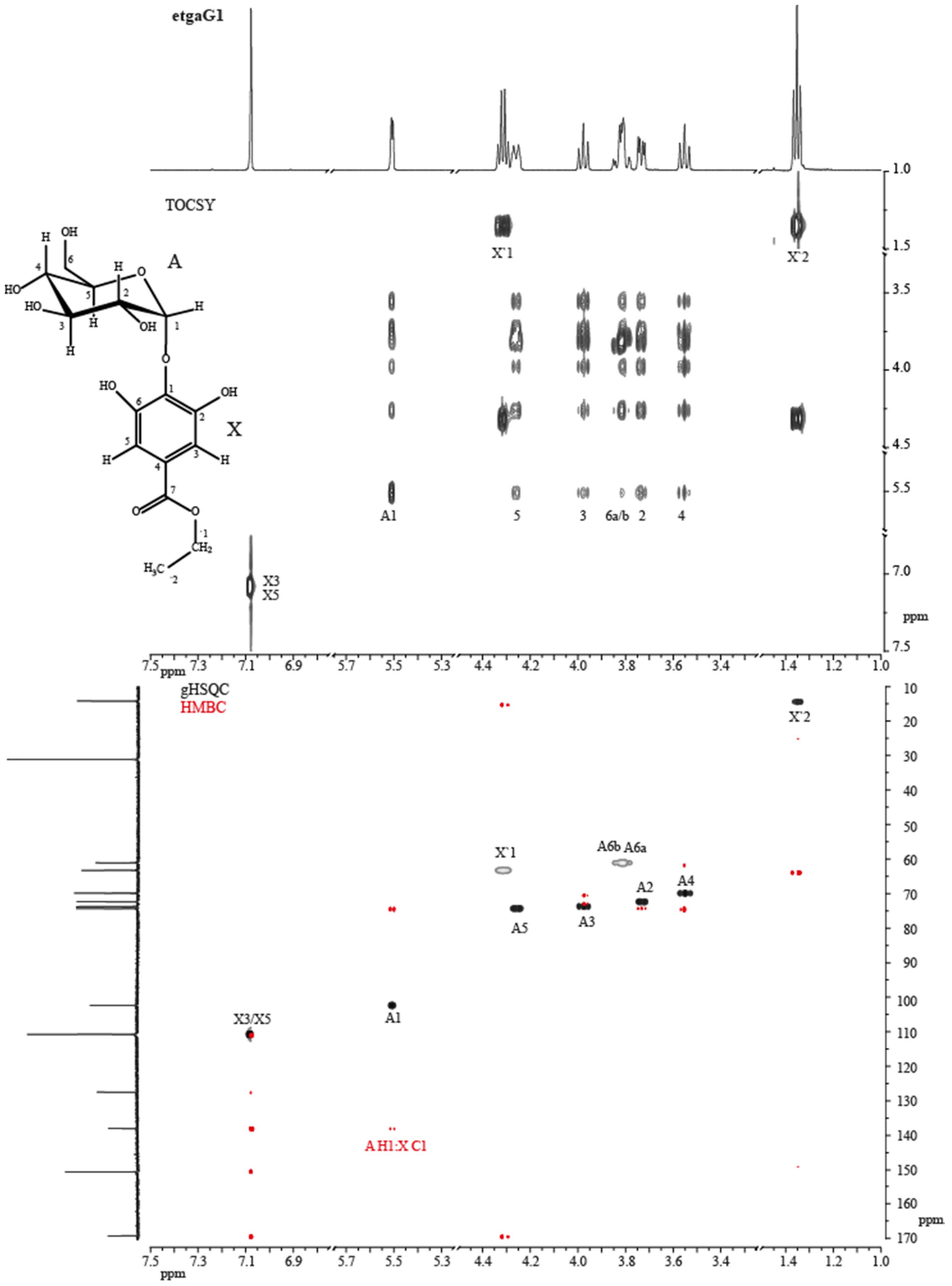

Fig. S4 1D ^{1}H NMR spectrum, 2D ^{1}H-^{1}H TOCSY (150 ms mixing time), 2D ^{13}C-^{1}H HSQC (black), and HMBC (red) spectra and 1D ^{13}C NMR spectrum of **etgaG1** structure. Peaks are marked in the spectrum, **A1** signifies H-1 and/or C-1 of glucose moiety **A**. Ethylgallate atoms are

marked by **X**1-7 for the gallic acid part and **X**`1-2 for the ethyl part. The relevant 3-bond HMBC correlation between **A** H-1 and **X** C-1 is marked. Peak numbers correspond with the atom numbers indicated in the structure

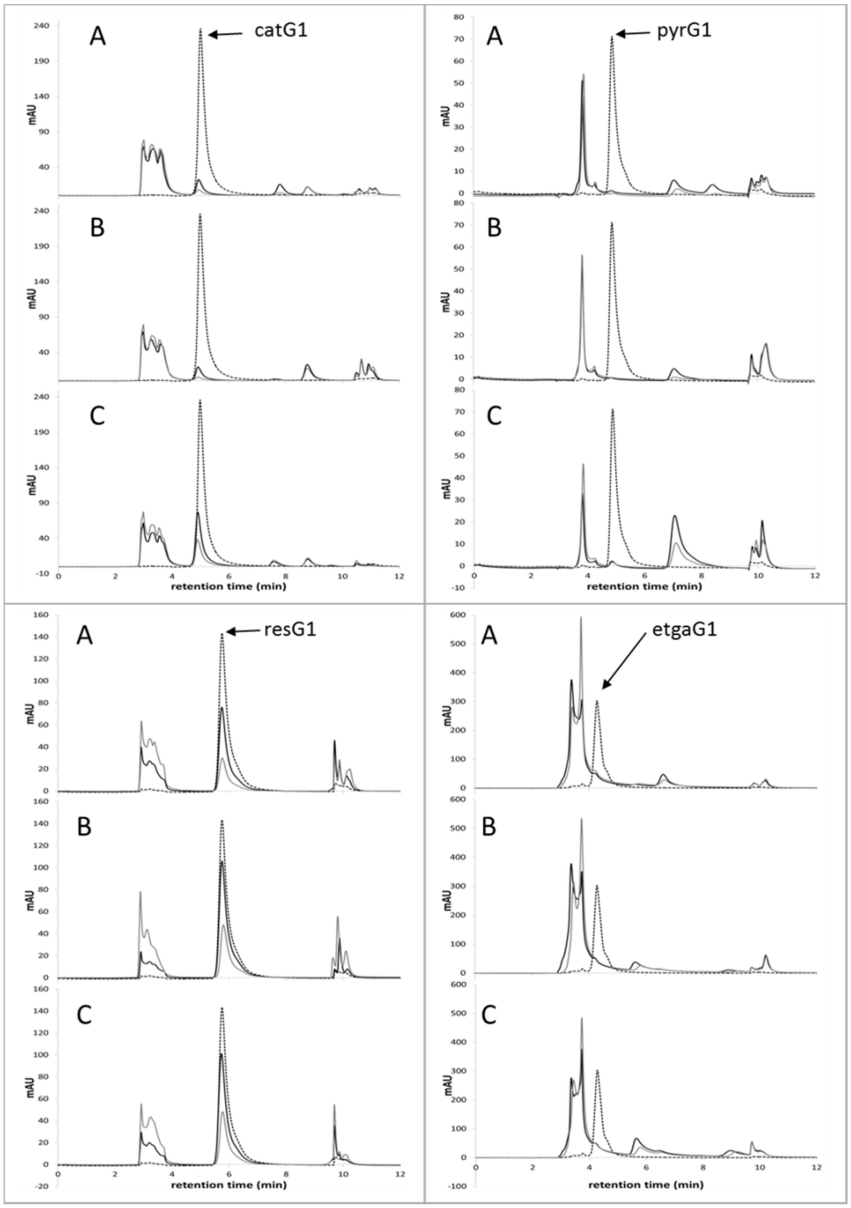

Fig. S5 NP-HPLC product profiles of an incubation of 1.25 mg/mL (A) GtfA-ΔN, (B) Gtf180-ΔN, and (C) GtfMLI-ΔN for t = 0 (dotted black line), 15 (gray line), and 60 (black line) min with 100 mM of the mono-glucosides catechol-α-D-glucoside (catG1), pyrogallol-α-D-glucoside (pyrG1), resorcinol-α-D-glucoside (resG1), and ethyl gallyl-α-D-glucoside (etgaG1)

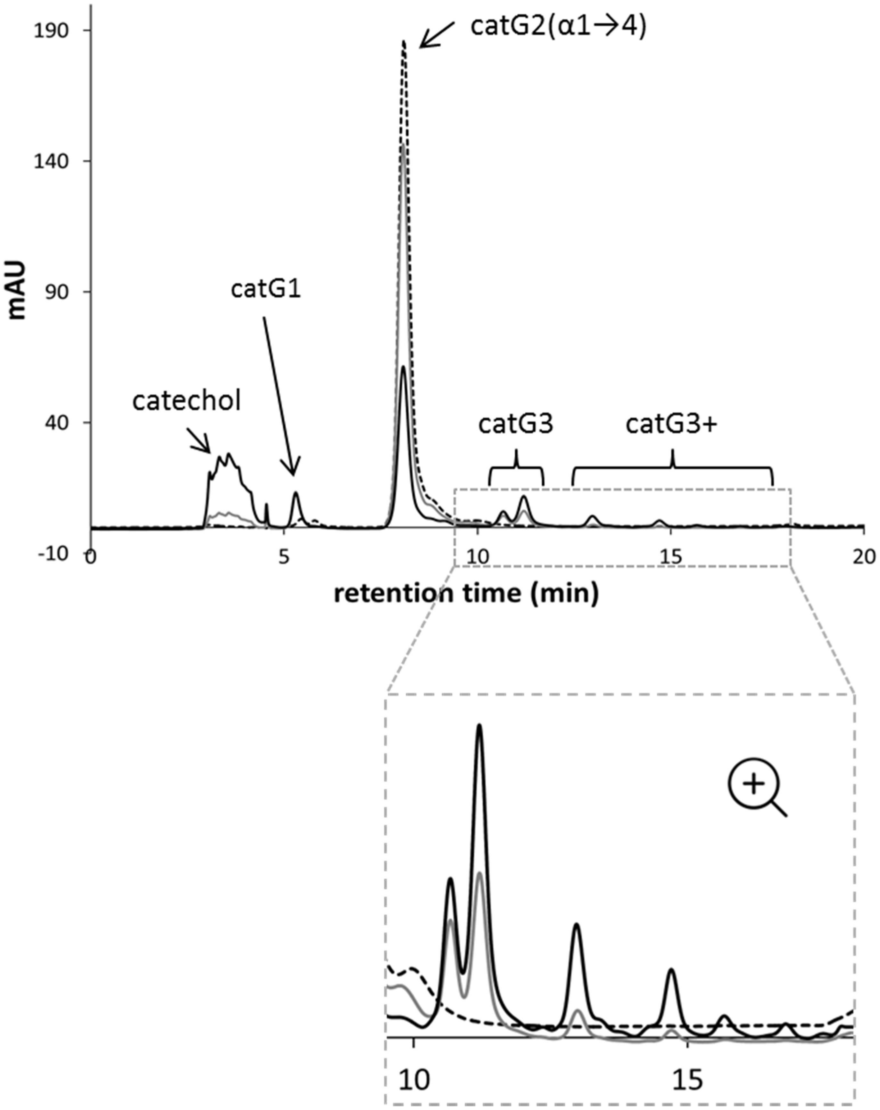

Fig. S6 NP-HPLC product profiles of an incubation of 1.25 mg/mL GtfA-ΔN for t = 0 (dotted black line), 15 (gray line), and 60 (black line) min with 100 mM cat^{4}G2. catG3 = catechol tri-glucoside; cat3G+ = catechol glucosides with more than 3 glucose moieties attached
